# The impact of atorvastatin on cardiometabolic risk factors in brothers of women with polycystic ovary syndrome

**DOI:** 10.1007/s43440-020-00135-w

**Published:** 2020-07-21

**Authors:** Robert Krysiak, Witold Szkróbka, Bogusław Okopień

**Affiliations:** Department of Internal Medicine and Clinical Pharmacology, Medical University of Silesia, Medyków 18, 40-752 Katowice, Poland

**Keywords:** Hypercholesterolemia, Polycystic ovary syndrome, Risk factors, Statins

## Abstract

**Background:**

Women with polycystic ovary syndrome (PCOS) are characterized by increased cardiometabolic risk. The aim of the current study was to compare the impact of atorvastatin on plasma levels of cardiometabolic risk factors between men whose sisters had either PCOS or were unaffected.

**Methods:**

The study population consisted of two age-, fat-free mass index-, blood pressure- and plasma lipid-matched groups of men with elevated total and LDL cholesterol levels: 20 brothers of PCOS probands (group 1) and 20 brothers of healthy women (group 2). Both groups were then treated with atorvastatin (40 mg daily) for the following 6 months. At the beginning and at the end of the study, we assessed plasma lipid levels, glucose homeostasis markers and levels of dehydroepiandrosterone sulfate, testosterone, bioavailable testosterone, uric acid, high-sensitivity C-reactive protein (hsCRP), homocysteine, fibrinogen and 25-hydroxyvitamin D.

**Results:**

At the beginning of the study, both treatment arms differed in the degree of insulin resistance, calculated bioavailable testosterone, as well as in plasma levels of dehydroepiandrosterone sulfate, uric acid, hsCRP and 25-hydroxyvitamin D. Although atorvastatin reduced total and LDL cholesterol levels, this effect was stronger in group 2 than group 1. In group 2, atorvastatin exerted also a more potent impact on hsCRP, fibrinogen and homocysteine. An unfavorable impact on insulin sensitivity was observed only in group 1; while, statistically significant changes in uric acid and 25-hydroxyvitamin D levels were found only in group 2.

**Conclusion:**

The obtained results suggest that cardiometabolic effects of atorvastatin are less pronounced in male siblings of PCOS probands than in brothers of unaffected women.

## Introduction

Polycystic ovary syndrome (PCOS) affects 6–20% of premenopausal women and seems to be the most common endocrine disorder in women of reproductive age [1,2]. In addition to chronic anovulation, hyperandrogenism and polycystic ovaries, the disorder is characterized by an increased prevalence of abdominal obesity, insulin resistance, deterioration of glucose metabolism, dyslipidemia and low-grade systemic inflammation [1,2]. PCOS also carries significant risk for the development of metabolic and cardiovascular complications, including diabetes, metabolic syndrome and cardiovascular disease [3,4].

The results of several studies are in favor of increased cardiometabolic risk also in male relatives of PCOS probands. Brothers of women with PCOS were more obese and more prone to central adiposity than unrelated healthy men [5]. Systolic, but not diastolic blood, pressure was significantly higher in male siblings of PCOS probands than in control men [5,6]. Men whose sisters were diagnosed with PCOS had increased levels of 2-h postchallenge glucose, were more insulin-resistant, more frequently met the criteria of metabolic syndrome [5,7–10], as well as were characterized by a higher prevalence of type 2 diabetes and impaired glucose tolerance than brothers of unaffected women [11]. They were also characterized by impaired brachial artery flow-mediated dilatation and this impairment was particularly pronounced in subjects with a family history of diabetes mellitus [9,12]. Circulating levels of adiponectin, but not of resistin and homocysteine, were lower in male siblings of PCOS probands than unrelated healthy control subjects [13]. Finally, brothers of women with PCOS had increased levels of plasminogen activator inhibitor-1 and factor VII [7]. Interestingly, compared with matched controls, male siblings of women with PCOS were found to have significantly elevated dehydroepiandrosterone-sulfate (DHEA-S) levels [9,10,14], as well as lower concentrations of bioavailable testosterone [5]. Recently, we have observed that exogenous testosterone potentiated lipid-lowering and extra-lipid effects of hypolipidemic agents in subjects with low testosterone levels [15,16]. Therefore, the aim of the current study was to compare the impact of atorvastatin on plasma levels of cardiometabolic risk factors between men whose sisters had either PCOS or were unaffected.

## Materials and methods

### Patients

The study population consisted of 40 adult men (aged 30–70 years) with elevated levels of total cholesterol (above 200 mg/dL) and low-density lipoprotein (LDL) cholesterol (more than 130 mg/dL), who had been following the lifestyle modification intervention for at least 12 weeks before entering the study. These subjects were screened at the outpatient clinic of our department for the presence of lipid abnormalities. Group 1 included 20 male siblings of women with PCOS (diagnosed based on the Rotterdam 2003 criteria). Group 2, serving as a control group, enrolled 20 male siblings of healthy adult women. These subjects were selected among hypercholesterolemic men by a computer algorithm, aimed at obtaining two groups matched for age, body mass index, blood pressure and plasma lipid levels. All participants were enrolled either in December or January (*n* = 21), or in June or July (*n* = 19) to limit the impact of seasonal fluctuations in the measured parameters.

Potential participants were excluded if they met any of the following criteria: cardiovascular disease (with the exception of mild arterial hypertension), diabetes, thyroid disorders, acute and chronic inflammatory processes, kidney or liver failure, malabsorption syndromes, as well as any pharmacotherapy.

All patients gave written informed consent before participating in the research after the nature of the study had been explained and each participant had the opportunity to ask questions. The study was conducted in accordance with the Declaration of Helsinki, and the protocol was approved by the local ethics committee.

### Study design

Over the entire study period (6 months), all men were treated with atorvastatin (40 mg daily), which was administered once daily at bedtime. No changes in treatment regimen were allowed. Throughout the study, all participants were also required to restrict intakes of total fat to less than 30% of energy intake, saturated fat to less than 7% of energy intake, and cholesterol to less than 200 mg per day, as well as were encouraged to increase fiber intake to 15 g per 1000 kcal. They were also recommended to do at least 150 min of moderate-intensity aerobic physical activity per week. Moderate-intensity activity was defined based on the rate of energy expenditure during the activity (between 3 and 6 metabolic equivalents) and on a person’s level of effort (5 or 6 on a scale of 0–10, where sitting is 0 and the highest level of effort is 10). The recommended activities included brisk walking (at least 4 km/h), water aerobics, slow dancing (ballroom or social), tennis (doubles), biking slower than 16 km/h, hiking, washing windows, sweeping the floor, vacuuming or pushing a lawn mower. The participants were also asked to do muscle-strengthening activities that were moderate intensity and involved all major muscle groups on two or more days a week. Compliance was assessed every 8 weeks by tablet counts and analysis of individual dietary questionnaires and diaries in which they continuously recorded all their activities.

### Anthropometric measurements

Body mass index was calculated as weight (in kg) divided by height (in m) squared. Fat-free mass was measured using bioelectrical impedance analysis (model BF-511 B, Omron Healthcare Europe, Hoofddorp, the Netherlands), which is based on different conductance and impedance of fat and fat-free tissue. Fat-free mass index was calculated with the following formula: fat-free mass/ height squared.

### Laboratory assays

Laboratory assays were performed in the first and in the last days of the study. All venous blood samples were collected between 7.30 and 8.30 a.m., after an overnight 12-h fasting and assayed in duplicate. Plasma concentrations of total cholesterol, LDL cholesterol, HDL cholesterol, triglycerides, glucose, creatinine and uric acid were measured with standard methods (Roche Diagnostics, Basel, Switzerland). Plasma levels of insulin, DHEA-S, total testosterone, sex hormone-binding globulin, high-sensitivity C-reactive protein (hsCRP), homocysteine and 25-hydroxyvitamin D were assayed using an electrochemiluminescent analyzer (Cobas e411, Roche Diagnostics, Mannheim, Germany). Fibrinogen was measured by the Clauss technique using an automated BCS XP analyzer (Siemens Healthcare, Warsaw, Poland). The homeostatic model assessment 1 of insulin resistance (HOMA1-IR) was calculated as fasting blood glucose (mg/dL)/*x* fasting insulin (mIU/L)/405. Bioavailable testosterone was calculated based on testosterone and sex hormone-binding globulin levels using the online calculator (https://www.issam.ch/freetesto.htm). The estimated glomerular filtration rate was calculated as follows: 175 × [plasma creatinine (µmol/L) × 0.0113]^−1.154^ × age (years)^0.203^ (the Modification Diet in Renal Disease Study equation).

### Statistical analysis

Before statistical analysis, all data were subjected to log-transformation to meet the criteria of normality and homogeneity. The study arms, as well as differences between percent changes from baseline after adjustment for baseline values (reflecting atorvastatin action), were compared using Student’s *t* test for independent samples after consideration of age, fat-free mass index, smoking and blood pressure as potential confounders. The differences between baseline and post-treatment values within the same study arm were analyzed with Student’s paired *t* test. Qualitative data were compared with *χ*^2^ tests. Associations between variables were tested using Pearson's correlation coefficient. Two-tailed *p* values below 0.05 were considered statistically significant.

## Results

At baseline, there were no statistically significant differences between groups 1 and 2 in age, body smoking, mass index, fat-free mass, fat-free mass index, blood pressure, as well as in plasma levels of total cholesterol, LDL cholesterol, HDL cholesterol, triglycerides, glucose, total testosterone, fibrinogen, homocysteine and the glomerular filtration rate. HOMA1-IR, DHEA-S, uric acid and hsCRP were higher, while calculated bioavailable testosterone and 25-hydroxyvitamin D were lower in group 1 than group 2 (Table 1).

**Table 1:** Baseline characteristics of patients

Variable	Group 1^a^	Group 2^b^	*p* value (group 1 vs. group 2)^c^
Number (*n*)	20	20	–
Age [years; mean (SD)]	50 (12)	52 (10)	0.5703
Smokers (%)	25	30	–
Body mass index [kg/m^2^; mean (SD)]	28.5 (4.8)	28.1 (5.0)	0.7977
Fat-free mass [kg; mean (SD)]	58.5 (5.9)	62.0 (6.5)	0.0826
Fat-free mass index [kg/m^2^; mean (SD)]	18.8 (2.6)	19.6 (2.0)	0.2823
Systolic blood pressure [mmHg; mean (SD)]	134 (12)	132 (15)	0.6441
Diastolic blood pressure [mmHg; mean (SD)]	85 (8)	84 (8)	0.6948
Total cholesterol [mg/dL; mean (SD)]	265 (37)	270 (30)	0.6414
LDL cholesterol [mg/dL; mean (SD)]	172 (22)	175 (21)	0.6616
HDL cholesterol [mg/dL; mean (SD)]	48 (11)	50 (12)	0.5859
Triglycerides [mg/dL; mean (SD)]	190 (31)	178 (35)	0.2582
Glucose [mg/dl; mean (SD)]	95 (14)	91 (16)	0.4054
HOMA1-IR [mean (SD)]	3.4 (1.0)	2.6 (0.8)	**0.0081**
DHEA-S [µmol/L; mean (SD)]	4.6 (0.8)	3.9 (0.7)	**0.0055**
Total testosterone [nmol/L; mean (SD)]	19.5 (5.5)	22.6 (7.6)	0.1477
Calculated bioavailable testosterone [nmol/L; mean (SD)]	8.4 (2.5)	10.6 (3.0)	**0.0161**
Uric acid [mg/dL; mean (SD)]	5.3 (1.6)	4.3 (1.1)	**0.0051**
hsCRP [mg/L; mean (SD)]	3.1 (0.7)	2.5 (0.7)*	**0.0100**
Homocysteine [μmol/L; mean (SD)]	26 (7)	28 (8)	0.4054
Fibrinogen [mg/dL; mean (SD)]	390 (80)	374 (72)	0.5102
25-Hydroxyvitamin D [mg/dL; mean (SD)]	25 (9)	32 (8)	**0.0132**
Estimated glomerular filtration rate [ml/min/1.73m^2^; mean (SD)]	86 (12)	88 (14)	0.6304

Bold values indicate statistical significance (*p* values)

^a^Male siblings of women with polycystic ovary syndrome

^b^Male siblings of healthy adult women

^c^Both groups were compared using Student’s *t* test for independent samples after consideration of age, fat-free mass index, smoking and blood pressure as potential confounders

Although all values were natural log transformed, the table shows the raw data because the mean and SD values of log-transformed data are less relevant. Two-tailed *p* values below 0.05 were considered statistically significant

Because no serious adverse effects were reported, all patients completed the follow-up and were included in the final analysis. Atorvastatin did not affect body mass index, fat-free mass and fat-free mass index. There were no differences between both groups in physical activity.

In both groups, atorvastatin reduced total and LDL cholesterol levels, as well as decreased hsCRP, homocysteine and fibrinogen. Moreover, in group 1, the drug increased HOMA1-IR; while in group 2, atorvastatin reduced uric acid and increased 25-hydroxyvitamin D levels. In both groups, atorvastatin exerted a neutral effect on plasma levels of HDL cholesterol, triglycerides, glucose, DHEA-S, testosterone and calculated bioavailable testosterone, as well as on the estimated glomerular filtration rate. The effect of atorvastatin on total cholesterol, LDL cholesterol, uric acid, hsCRP, homocysteine, fibrinogen and 25-hydroxyvitamin D was stronger; while, the impact on HOMA1-IR was less expressed in group 2 than group 1. At the end of the study period, there were differences between the treatment arms in total cholesterol, LDL cholesterol, HOMA1-IR, DHEA-S, calculated bioavailable testosterone, uric acid, hsCRP, homocysteine, fibrinogen and 25-hydroxyvitamin (Table 2).

**Table 2:** The effect of atorvastatin treatment on plasma lipids, glucose homeostasis markers, hormones and the investigated cardiovascular risk factors in male siblings of women with and without polycystic ovary syndrome

Variable	Group 1^a^	Group 2^b^	*p* value (group 1 vs. group 2)^c^
Total cholesterol [mg/dl; mean (SD)]			
At the beginning of the study	265 (37)	270 (30)	0.6414
At the end of the study	223 (32)	197 (29)^e^	**0.0105**
*p* value [post-treatment vs. baseline]^d^	**0.0005**	**< 0.0001**	–
LDL cholesterol [mg/dL; mean (SD)]			
At the beginning of the study	172 (22)	175 (21)	0.6616
At the end of the study	131 (19)	104 (17)^e^	**< 0.0001**
*p* value [post-treatment vs. baseline]^d^	**< 0.0001**	**< 0.0001**	–
HDL cholesterol [mg/dL; mean (SD)]			
At the beginning of the study	48 (11)	50 (12)	0.5859
At the end of the study	52 (11)	56 (14)	0.3214
*p* value [post-treatment vs. baseline^d^	0.2574	0.1538	–
Triglycerides [mg/dL; mean (SD)]			
At the beginning of the study	190 (31)	178 (35)	0.2582
At the end of the study	176 (34)	160 (40)	0.1809
*p* value [post-treatment vs. baseline]^d^	0.1816	0.1382	-
Glucose [mg/dl; mean (SD)]			
At the beginning of the study	95 (14)	91 (16)	0.4054
At the end of the study	99 (11)	92 (12)	0.0620
*p* value [post-treatment vs. baseline]^d^	0.3214	0.8243	–
HOMA1-IR [mean (SD)]			
At the beginning of the study	3.4 (1.0)	2.6 (0.8)	**0.0081**
At the end of the study	4.1 (0.8)^e^	2.7 (0.7)	**< 0.0001**
*p* value [post-treatment vs. baseline]^d^	**0.0193**	0.6763	–
DHEA-S [µmol/L; mean (SD)]			
At the beginning of the study	4.6 (0.8)	3.9 (0.7)	**0.0055**
At the end of the study	4.4 (0.9)	3.7 (0.8)	**0.0132**
*p* value [post-treatment vs. baseline]^d^	0.4622	0.4054	–
Total testosterone [nmol/L; mean (SD)]			
At the beginning of the study	19.5 (5.5)	22.6 (7.6)	0.1477
At the end of the study	18.7 (6.0)	21.9 (7.2)	0.1351
*p* value [post-treatment vs. baseline]^d^	0.6628	0.7665	–
Calculated bioavailable testosterone [nmol/L; mean (SD)]			
At the beginning of the study	8.4 (2.5)	10.6 (3.0)	**0.0161**
At the end of the study	8.3 (2.0)	10.3 (2.6)	**0.0096**
*p* value [post-treatment vs. baseline]^d^	0.8896	0.7372	–
Uric acid [mg/dL; mean (SD)]			
At the beginning of the study	5.3 (1.6)	4.3 (1.1)	**0.0051**
At the end of the study	4.8 (1.2)	3.4 (0.8)^e^	**0.0001**
*p* value [post-treatment vs. baseline]^d^	0.2706	**0.0053**	–
hsCRP [mg/L; mean (SD)]			
At the beginning of the study	3.1 (0.7)	2.5 (0.7)	**0.0100**
At the end of the study	2.5 (0.6)	1.3 (0.6)^e^	**< 0.0001**
*p* value [post-treatment vs. baseline]^d^	**0.0060**	**< 0.0001**	–
Homocysteine [μmol/L; mean (SD)]			
At the beginning of the study	26 (7)	28 (8)	0.4054
At the end of the study	21 (6)	16 (6)^e^	**0.0121**
*p* value [post-treatment vs. baseline]^d^	**0.0202**	**< 0.0001**	**–**
Fibrinogen [mg/dL; mean (SD)]			
At the beginning of the study	390 (80)	374 (72)	0.5102
At the end of the study	329 (67)	278 (70)^e^	**0.0239**
*p* value [post-treatment vs. baseline]^d^	**0.0127**	**0.0001**	–
25-Hydroxyvitamin D [mg/dL; mean (SD)]			
At the beginning of the study	25 (9)	32 (8)	**0.0132**
At the end of the study	26 (8)	40 (9)^e^	**< 0.0001**
*p* value [post-treatment vs. baseline]^d^	0.7124	**0.0051**	–
Estimated glomerular filtration rate [ml/min/1.73m^2^; mean (SD)]			
At the beginning of the study	86 (12)	88 (14)	0.6304
At the end of the study	88 (15)	85 (13)	0.5032
*p* value [post-treatment vs. baseline]^d^	0.6441	0.4868	–

Bold values indicate statistical significance (*p* values)

Although all values were natural log transformed, the table shows the raw data because the mean and SD values of log-transformed data are less relevant. Two-tailed *p* values below 0.05 were considered statistically significant

^a^Male siblings of women with polycystic ovary syndrome

^b^Male siblings of healthy adult women

^c^Both groups were compared using Student’s *t* test for independent samples after consideration of age, fat-free mass index, smoking and blood pressure as potential confounders

^d^Differences between post-treatment and baseline values were identified using Student’s paired *t* test

^e^The impact of atorvastatin (percent changes from baseline after adjustment for baseline values) stronger than in the second group (comparisons were performed using Student’s *t* test for independent samples after consideration of age, fat-free mass index, smoking and blood pressure as potential confounders)

At baseline, plasma concentrations of uric acid, hsCRP, homocysteine and fibrinogen correlated with plasma levels of total cholesterol [group 1: *r* values between 0.25 (*p* = 0.0493) and 0.37 (*p* = 0.0074); group 2: *r* values between 0.32 (*p* = 0.0231) and 0.47 (*p* = 0.0002)] and LDL cholesterol (group 1: *r* values between 0.26 (*p* = 0.043) and 0.41 (*p* = 0.0004)] group 2: *r* values between 0.34 (*p* = 0.0180) and 0.49 (*p* = 0.0001)]. There were also correlations between total and LDL cholesterol and 25-hydroxyvitamin D [group 1: *r* = − 0.35 (*p* = 0.0110) and *r* = − 0.38 (*p* = 0.0095); group 2: *r* = − 0.40 (*p* = 0.0008) and *r* = − 0.43 (*p* = 0.0002)], as well as between baseline HOMA1-IR and baseline values of uric acid [group 1: *r* = 0.40 (*p* = 0.0005); group 2: *r* = 0.49 (*p* < 0.0001)], CRP [group 1: *r* = 0.37 (*p* = 0.0068); group 2: *r* = 0.39 (*p* = 0.0001)] and 25-hydroxyvitamin D [group 1: *r* = − 0.32 (*p* = 0.0325); group 2: *r* = − 0.34 (*p* = 0.0285)]. The impact of atorvastatin on uric acid, hsCRP, fibrinogen, homocysteine and 25-hydroxyvitamin D correlated with baseline calculated bioavailable testosterone levels [group 1: *r* values between − 0.26 (*p* = 0.0415) and − 0.35 (*p* = 0.0092); group 2: *r* values between − 0.34 (*p* = 0.0012) and − 0.49 (*p* < 0.0001)] but did not correlate with its action on plasma lipids. Treatment-induced changes in uric acid, hsCRP, fibrinogen and homocysteine inversely correlated with baseline 25-hydroxyvitamin D levels [group 1: *r* values between − 0.24 (*p* = 0.046) and − 0.34 (*p* = 0.0355); group 2: *r* values between − 0.31 (*p* = 0.0261) and − 0.42 (*p* = 0.0002)], and in group 2 with treatment-induced increase in 25-hydroxyvitamin D [*r* values between 0.28 (*p* = 0.0295) and 0.38 (*p* = 0.0025)]. In group 1, atorvastatin-induced increase in HOMA1-IR correlated with its baseline value [*r* = − 0.47 (*p* = 0.0001)]. No other correlations were significant.

## Discussion

The current study has shown for the first time that male siblings of PCOS probands were characterized by abnormal levels of some of factors known to be involved in the pathogenesis of atherosclerosis and carbohydrate disorders and to predict the development of cardiovascular disease, diabetes and metabolic syndrome [17–22], as well as that hypolipidemic and pleiotropic effects of atorvastatin were less pronounced in brothers of PCOS probands than in unrelated matched control men. Although at baseline both groups differed in HOMA1-IR and in plasma levels of uric acid, hsCRP and 25-hydroxyvitamin D, the extent of these abnormalities was less pronounced than observed previously in women with PCOS [23]. Moreover, both groups differed in androgen levels. Owing to preselection resulting in similar baseline levels, the obtained results cannot be explained by differences in body mass index, fat-free mass, blood pressure or plasma lipids. Moreover, strict inclusion and exclusion criteria minimized the possible impact of other disorders or concomitant therapies.

Based on the obtained results, some clinical conclusions can be drawn. First, our findings are in line with the view that genetic factors play an important role in etiology of PCOS [24]. Second, male brothers of women with PCOS syndrome may be more prone to the development and progression of metabolic and cardiovascular disorders, especially if they have concomitant risk factors. However, relative cardiovascular risk in male siblings of women with PCOS seems to be lower than in probands. Third, male siblings of women with PCOS are probably less likely to benefit or benefit to a lesser degree from statin therapy than their unrelated peers. Finally, less expressed benefits of atorvastatin treatment in those men may be partially associated with abnormal testosterone production.

Male siblings of women with PCOS were characterized by higher DHEA-S levels, as well as lower values of calculated bioavailable testosterone than their counterparts without a family history of this disorder. Bioavailable testosterone calculated by Vermeulen’s formula, used in the present study, reflects the sex hormone-binding protein-unbound fraction of this hormone and correlates well with free testosterone assessed by equilibrium dialysis [25]. Therefore, small differences in testosterone production between both arms, as in the current study, may be reflected by differences in bioavailable testosterone despite similar levels of total hormone. The lack of fluctuations and long half-life cause that DHEA-S reflects well adrenal production of dehydroepiandrosterone [26], which is a precursor of sex steroid hormones and has the potential to be converted to testosterone [26]. Therefore, increased DHEA-S levels and decreased values of calculated bioavailable testosterone in brothers of PCOS probands suggest that they may be characterized by impaired conversion of dehydroepiandrosterone to testosterone. It seems that abnormal production of testosterone rather than of dehydroepiandrosterone contributes to increased cardiometabolic risk in male siblings of PCOS. In line with this conclusion, baseline and treatment-induced changes in levels of all measured risk factors correlated with calculated bioavailable testosterone but not with DHEA-S. Interestingly, between-group differences in testosterone were discrete and this finding may explain why attenuating effect on atorvastatin action was less pronounced in brothers of PCOS probands that in men with late-onset hypogonadism participating in the previous study [15]. In both treatment arms, atorvastatin exerted a neutral effect on total and calculated bioavailable testosterone, which is line with previous observations that only aggressive statin therapy, reducing cholesterol levels to subnormal values, impairs testicular steroidogenesis [27].

Despite numerous cardiovascular benefits associated with statin therapy in subjects with impaired glucose homeostasis, 3-hydroxy-3-methylglutaryl coenzyme A (HMG-CoA) reductase inhibitors slightly increase the risk of type 2 diabetes, probably because exerting an unfavorable effect on insulin sensitivity and secretion [28]. Both study arms, which included only diabetes-free men, differed in baseline values of HOMA1-IR and this finding is in line with the previous observations indicating that male siblings of PCOS probands are characterized by impaired insulin sensitivity [5,7–10]. The presence of correlation between baseline HOMA1-IR and baseline values of uric acid, hsCRP and 25-hydroxyvitamin D indicates that the increased cardiometabolic risk in these subjects is partially associated with disturbed insulin action. Moreover, unlike control subjects, atorvastatin administered to brothers of women with PCOS worsened insulin sensitivity, which correlated with baseline HOMA1-IR. This finding suggests that the unfavorable effect of HMG-CoA reductase inhibitor therapy on glucose homeostasis in male siblings of PCOS probands may be secondary to their more atherogenic metabolic profile.

The impact of atorvastatin on uric acid, hsCRP, fibrinogen, homocysteine and 25-hydroxyvitamin D did not correlate with treatment-induced changes in total and LDL cholesterol, suggesting its pleiotropic nature. Cardiometabolic effects of atorvastatin may be associated with the inhibitory impact on protein prenylation, nuclear factor-κB pathway and leukocyte function-associated antigen-1-intercellular adhesion molecule-1 interaction [29,30]. Interestingly, PCOS is associated with the activation of intranuclear nuclear factor-κB [31], as well as with the increased expressions of leukocyte function-associated antigen-1 and intercellular adhesion molecule-1 [32]. Moreover, extra-lipid effects of statin therapy in women with PCOS, mediated probably by the inhibitory effects on farnesylation and geranylgeranylation, seem to be weak [33]. If these mechanisms are disturbed also in brothers of women with PCOS, they may be more resistant to statin therapy as it was observed in our study.

The impact of atorvastatin on uric acid, hsCRP, fibrinogen and homocysteine inversely correlated with baseline 25-hydroxyvitamin D levels, and in subjects with a negative family history of PCOS also with treatment-induced increase in 25-hydroxyvitamin D. Interestingly, cholecalciferol is produced from 7-dehydrocholesterol, which is also a precursor for cholesterol [34] and the enhanced production of vitamin D may exert an inhibitory effect on the mevalonate pathway. Alternatively, taking into account the inhibitory effect of vitamin D on the nuclear factor κB signaling pathway [35], the improvement in vitamin D status only in one treatment arm may partially explain between-group differences in the strength of cardiometabolic effects of atorvastatin in our study.

Several study limitations need to be acknowledged. Because of the small sample size and short-term nature, this research should be regarded as a preliminary report. The current study did not investigate hard clinical endpoints, such as morbidity and mortality rates. It remains to be elucidated whether the obtained results represent a class effect of HMG-CoA reductase inhibitors or are associated with unique properties of atorvastatin. Finally, the study did not compare lipid and pleiotropic effects of atorvastatin between brothers of women with various phenotypes of PCOS.

In summary, compared with brothers of unaffected women, male siblings of women with PCOS were more insulin resistant, as well as had higher levels of uric acid, hsCRP and lower levels of 25-hydroxyvitamin D. In addition to differences in hypolipidemic effects, the impact of atorvastatin on cardiometabolic risk factors was more pronounced in subjects not having sisters with PCOS. The obtained results suggest that cardiometabolic effects of atorvastatin are less expressed in male siblings of women with PCOS. Because of study limitations, this issue needs further and deeper investigation in future studies with larger sample sizes.

## Abbreviations

DHEA-S: Dehydroepiandrosterone-sulfate
HDL: High-density lipoproteins
HMG-CoA: 3-Hydroxy-3-methylglutaryl coenzyme A
HOMA1-IR: The homeostasis model assessment 1 of insulin resistance index
hsCRP: High-sensitivity C-reactive protein
LDL: Low-density lipoproteins
PCOS: Polycystic ovary syndrome
SD: Standard deviation
